# Cellular strategies for repairing trapped protein-DNA complexes

**DOI:** 10.3389/fphar.2026.1716415

**Published:** 2026-02-10

**Authors:** Maria Sideridou, Doukissa Ioanna Machli, Dora Lontra, Theodoros Rampias

**Affiliations:** 1 Biomedical Research Foundation, Academy of Athens, Athens, Greece; 2 School of Medicine and Health Sciences, Keele Greece University, Athens, Greece

**Keywords:** cancer, chemotherapy, DNA repair, PARP inhibitors, protein-DNA crosslinks, topoisomerase poisons

## Abstract

DNA-protein crosslinks (DPCs) are highly toxic DNA lesions that arise both from normal cellular metabolism and as an intended consequence of cancer chemotherapy. Key anticancer agents, including topoisomerase poisons and PARP inhibitors, exert their therapeutic effects by trapping enzymes on DNA, converting them into toxic barriers that block replication. To counteract this threat, cells have evolved specialized mechanisms to detect and remove DPCs. This review explores the molecular mechanisms by which these therapies trap proteins on DNA and the multi-layered defense systems cells use to resolve them-ranging from enzymatic degradation to mechanical extraction. We further examine how these processes are modulated by the cell cycle and chromatin landscape. Importantly, we highlight emerging evidence that alterations in DPC repair pathways are frequent in cancer and serve as critical determinants of treatment response. Ultimately, this review integrates mechanistic insights with clinical data to highlight how exploiting DPC repair defects can overcome drug resistance and guide the development of rational, synthetic lethal combination therapies.

## 1 Introduction

Among the diverse types of DNA damage, DNA-protein crosslinks (DPCs) represent a uniquely toxic and structurally complex class of lesions, as they physically obstruct essential DNA transactions such as replication and transcription. In oncology, DPCs have gained particular importance because several cornerstone chemotherapeutic agents deliberately induce trapped proteins-DNA complexes as a primary mechanism of cytotoxicity. Despite their central role in cancer therapy, the study of DPCs has historically been fragmented. Much of the existing literature has focused on individual drug classes, such as topoisomerase poisons or PARP inhibitors, or on isolated repair factors, without integrating these findings into a unified framework that explains how diverse trapped protein complexes are processed by the cell and how these processes influence therapeutic response, resistance, and toxicity. Consequently, the broader pharmacological and clinical implications of DPC repair have remained underappreciated.

This gap has become increasingly evident with recent advances that have fundamentally reshaped the field. The discovery of specialized DPC repair mechanisms-including DNA-dependent proteases, ATP-driven protein extraction systems, and highly coordinated post-translational signaling networks-has revealed that DPC repair is a tightly regulated and multi-layered defence system rather than a passive repair pathway. Importantly, alterations in these pathways are now recognized as recurrent features of human cancers and as critical determinants of sensitivity to DPC-inducing agents. At the same time, emerging pharmacological strategies directly targeting DPC repair components have highlighted the therapeutic potential of exploiting these pathways of cancer treatments, making a comprehensive and timely synthesis of the field necessary.

In this review, we provide an integrated overview of the molecular origins of DPCs, their deliberate induction by anticancer therapies, and the cellular strategies that resolve these lesions. We first discuss the chemical and biological mechanisms that give rise to DNA-protein crosslinks, followed by a detailed examination of the major DPC repair pathways, including proteolytic debulking, mechanical extraction, direct hydrolysis, and nucleolytic excision. We then explore how DPC repair is modulated by cell cycle progression and chromatin context, and how defects in these pathways contribute to cancer development, treatment response, and therapeutic resistance. Finally, we highlight emerging opportunities to target DPC repair mechanisms as a strategy for rational combination therapies and personalized cancer treatment.

### 1.1 Abasic sites as a nexus for DNA-protein crosslink formation

The integrity of the genome is under constant threat from both endogenous and exogenous sources of DNA damage. Such damage encompasses a broad spectrum of lesions, including abasic sites, mismatched or chemically modified bases (caused by alkylation or oxidation), single- and double-strand breaks, and covalent crosslinks. Crosslinks may occur either between bases on the same strand (intrastrand) or between opposite strands of the DNA helix (interstrand), each posing distinct challenges to replication and repair mechanisms (Weickert and Stingele, 2022).

An abasic (AP) site is a common DNA lesion that arises when a purine or pyrimidine base is lost, leaving the sugar–phosphate backbone intact. In normal cells, AP sites occur spontaneously through hydrolytic depurination or depyrimidination or, more frequently, as a key intermediate of the Base Excision Repair (BER) pathway following the enzymatic removal of a damaged base by a DNA glycosylase (Amidon and Eichman, 2020). The constant formation of AP sites represents a major endogenous threat to the genome’s integrity. If left unrepaired, these non-coding lesions can lead to mutations during DNA replication, as the cellular machinery may insert an incorrect base opposite the gap. This underscores the critical importance of the Base Excision Repair (BER) pathway, which constantly works to find and fix these AP sites to maintain genomic stability (Baute and Depicker, 2008). Cancer cells contain a significantly higher number of abasic sites, largely driven by oxidative and replication stress. Their hyperactive metabolism generates an excess of reactive oxygen species (ROS). These ROS can damage DNA in two ways: they may oxidize bases (creating lesions like 8-oxo-guanine that are removed to leave an AP site), or they may attack the backbone directly, causing the base to fall off. Furthermore, single-stranded DNA becomes exposed during replication; this fragile state makes it prone to spontaneous base loss, meaning replication stress further accelerates AP site formation in cancer cells (Lindahl, 1993).

Furthermore, metabolic rewiring in cancer cells often results in higher concentrations of reactive aldehydes that can react with DNA bases to form unstable adducts. The chemical instability of these adducts weakens the N-glycosidic bond, facilitating its cleavage and increasing the cellular context of AP sites (Lindahl, 1993).

**FIGURE 1:**
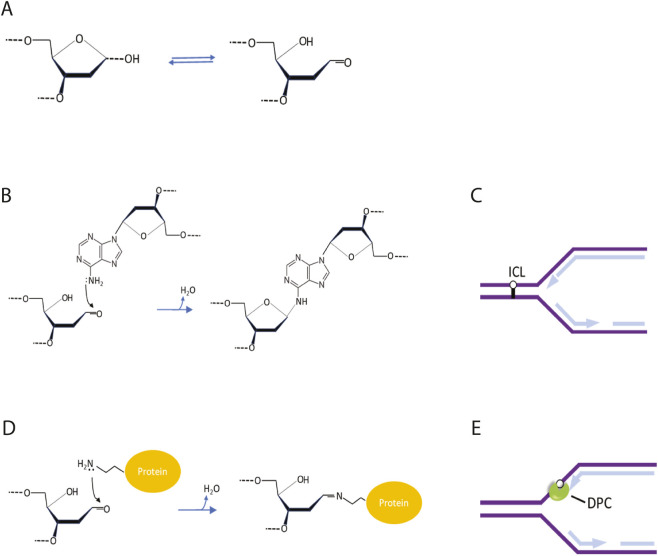
Formation of Trapped Protein Complexes and Interstrand Crosslinks at Abasic Sites. Schematic representation of the chemical reactions at an abasic (AP) site that lead to the formation of replication-blocking lesions. **(A)** An AP site exists in a chemical equilibrium between its stable, cyclic hemiacetal (furanose) form and its open-chain aldehyde form. This aldehyde group is highly electrophilic and reactive. **(B)** The aldehyde is susceptible to nucleophilic attack by the exocyclic primary amine of a base on the complementary strand (e.g., adenine), resulting in the formation of an interstrand crosslink (ICL). **(C)** An ICL covalently links the two DNA strands, creating a physical barrier that blocks the progression of replicative DNA polymerases. **(D)** Similarly, the AP site aldehyde can be attacked by a nucleophilic primary amine on a proximal protein, such as the ε-amino group of a lysine residue, to form a covalent DNA-protein crosslink (DPC), a type of trapped protein complex. **(E)** A DPC acts as a bulky lesion that sterically obstructs the DNA template, causing replication fork stalling and collapse if unrepaired.

Their accumulation is considered to lead to a high rate of point mutations during DNA replication increasing the genomic instability levels of cancer cells. In addition to causing point mutations, the inherent chemical reactivity of an AP site can generate more complex, replication-blocking damage such as covalent interstrand crosslinks (ICLs) or DNA-protein crosslinks (DPCs), both of which physically obstruct DNA replication machinery. Figure 1 illustrates the chemical progression from abasic sites to trapped complexes, highlighting how these endogenous lesions evolve into replication-blocking barriers structurally similar to those deliberately induced by chemotherapeutic agents. The chemical basis for the protein–DNA adducts formed by APs lies in the unique structure of the abasic site itself. The ability of AP sites to form these protein adducts stems from their unique chemical structure. An AP site fluctuates between a stable, closed ring form and a less common-but highly reactive-open aldehyde form. This open form acts as a chemical trap. It is strongly attracted to specific parts of proteins, particularly the lysine residues found in amino acid side chains. When they interact, they form a strong covalent bond that locks the protein to the DNA backbone, creating a DPC. Proteins that work closely with DNA, such as histones and polymerases, are especially prone to this trap. Once caught, they can stall replication forks, potentially leading to fork collapse and severe double-strand breaks s (Amidon and Eichman, 2020). In essence, the accumulation of abasic sites in cancer cells creates a reservoir of reactive aldehyde groups that can covalently trap nearby proteins, generating toxic DNA-protein crosslinks that impede replication.

### 1.2 Clinical application and development of protein-trapping drugs

The deliberate stabilization of transient enzyme-DNA intermediates into cytotoxic DNA-protein crosslinks (DPCs) represents a cornerstone of modern cancer chemotherapy, reflecting a pharmacological shift from inhibiting an enzyme’s catalytic activity to exploiting the genotoxic potential of the trapped enzymatic complex itself. Topoisomerases (TOPs) are exceptionally effective targets for this strategy due to their essential role in managing DNA topology in rapidly proliferating cancer cells.

To maintain genomic integrity during replication and transcription, topoisomerases resolve topological challenges such as DNA supercoiling. Topoisomerase I (TOP1) achieves this by inducing a transient single-strand break in the DNA. It forms a covalent bond between a catalytic tyrosine residue and the 3′-phosphate end of the nicked strand, creating a short-lived intermediate known as a cleavage complex. This allows the intact strand to rotate around the break, relieving torsional stress, after which TOP1 re-ligates the DNA backbone to restore its integrity. Topoisomerase II (TOP2), in contrast, creates a transient double-strand break and forms covalent bonds with both 5′-phosphate ends. It then passes an intact DNA duplex through the break before re-ligating the cleaved strands (Pommier, 2006).

This elegant catalytic cycle, however, can be lethally subverted by anticancer agents known as topoisomerase poisons. These small molecules do not bind to the enzyme or DNA alone but instead intercalate into the transient TOP-DNA cleavage complex. By physically obstructing the enzyme’s ability to re-ligate the cleaved DNA backbone, these agents convert the fleeting, physiological intermediate into a persistent, covalent DPC. When a replication fork collides with this stable DPC, the single- or double-strand breaks are transformed into permanent lesions, triggering cell cycle arrest and apoptosis (Perry and Ghosal, 2022; Kojima and Machida, 2020; Ruggiano and Ramadan, 2021).

The potential of this strategy was confirmed by identifying TOP2 as the target for drugs like etoposide and doxorubicin, and later, TOP1 as the target for the natural compound camptothecin (CPT). First isolated from plants over 60 years ago, CPT remains a milestone discovery (Li et al., 2017). Although thousands of derivatives were synthesized, only irinotecan and topotecan gained widespread approval, thanks to their better solubility and manageable toxicity (Garcia-Carbonero and Supko, 2002). Today, topoisomerase poisons are vital in oncology. They treat solid tumors such as colorectal, ovarian, and small-cell lung cancers, and are key components in therapies for hematological malignancies (like leukemias and lymphomas) and pediatric cancers (Dancey and Eisenhauer, 1996). The ongoing development of this drug class is evident in the thousands of clinical trials currently underway, all aiming to optimize efficacy and overcome resistance (Bondarev et al., 2024).

Poly (ADP-ribose) polymerase (PARP) inhibitors represent another important group of drugs that take advantage of the genotoxic potential of the trapped enzymatic complex itself. Poly (ADP-ribose) polymerases (PARPs) are a family of nuclear enzymes essential for maintaining genomic integrity through their central role in the DNA Damage Response (DDR). PARP1, the most abundant and well-characterized member involved in this process, facilitates the efficient resolution of single-strand breaks (SSBs) by the synthesis of branching chains of poly (ADP-ribose) (PAR) that are covalently attached to itself and other acceptor proteins. This PAR scaffold acts as a recruitment platform, orchestrating the rapid assembly of downstream repair complexes to promote efficient lesion resolution.

The therapeutic rationale for PARP inhibitors is founded upon the principle of synthetic lethality, which exploits the pre-existing genetic vulnerability of tumors with Homologous Recombination Deficiency (HRD) (Lord and Ashworth, 2017). HRD, often resulting from germline or somatic mutations in genes of the Homologous Recombination (HR) pathway, impairs the high-fidelity repair of DNA double-strand breaks (DSBs) (Helleday, 2011).

Pharmacological PARP inhibition prevents the efficient repair of endogenous single-strand breaks (SSBs). Subsequently, during S-phase, these unrepaired lesions trigger replication fork collapse and formation of double-strand breaks (DSBs) (Lord and Ashworth, 2017). In HR-deficient cancer cells, the inability to repair these drug-induced DSBs leads to extensive genomic fragmentation and subsequent apoptosis. This synthetic lethal interaction forms the basis of therapy for HRD-positive malignancies, with several PARP inhibitors approved for the treatment of advanced ovarian, breast, prostate, and pancreatic carcinomas (Lord and Ashworth, 2017).

First-generation compounds, developed in the 1970s, were nicotinamide analogues with only modest inhibitory activity (Bondar and Karpichev, 2024). A significant advance was achieved with second-generation inhibitors based on quinazoline-scaffold pharmacophores. The incorporation of a carboxamide group into these structures enhanced both affinity and specificity by forming strong hydrogen bonds within the PARP1 catalytic active site (Bondar and Karpichev, 2024; Jagtap and Szabo, 2005).

The third generation of PARP1 inhibitors (olaparib, niraparib, rucaparib, and talazoparib) introduced a dual mechanism of action, promoting PARP trapping in addition to catalytic inhibition (Curtin and Szabo, 2020). Acting as NAD + mimetics, they occupy the enzyme’s binding pocket to block the synthesis of poly (ADP-ribose). Crucially, binding also triggers a deep allosteric change in the PARP1 protein. This change increases the affinity of the DNA-binding domain for the strand break, effectively locking the entire enzyme in its DNA-bound conformation (Murai et al., 2012). The PARP inhibitor talazoparib is considered the most potent PARP trapper, exhibiting trapping efficiency several orders of magnitude greater than earlier compounds. This superior activity is associated with significant clinical efficacy in homologous recombination-deficient (HRD)-positive breast, ovarian, prostate, and pancreatic cancers, highlighting the critical cytotoxic role of the trapped PARP1-DNA complex (Hopkins et al., 2019; Litton et al., 2018). Thus, the therapeutic efficacy of both topoisomerase poisons and PARP inhibitors fundamentally relies on converting these essential enzymes into toxic, chromatin-bound obstacles that persist long enough to induce lethal replication stress.

### 1.3 Beyond topoisomerases and PARP: other trapped protein complexes in therapy

The principle of converting a cellular enzyme into a cytotoxic DNA–protein crosslink extends beyond topoisomerases and PARP1. Several other classes of drugs exploit distinct mechanisms to generate therapeutically relevant DPCs. One of the most clinically relevant examples involves the trapping of DNA methyltransferases (DNMTs). DNMT1, DNMT3A, and DNMT3B are responsible for adding methyl groups to cytosine residues in DNA. DNMT1 and DNMT3B cooperate to maintain DNA methylation and gene silencing in cancer cells, providing compelling evidence that DNA methylation is an essential step for neoplastic proliferation (Rhee et al., 2002).

The cytosine analogues 5-azacytidine (azacytidine) and 2′-deoxy-5-azacytidine (decitabine) are currently the most advanced drugs for epigenetic cancer therapy. Both agents have demonstrated significant clinical benefit in the treatment of myelodysplastic syndrome (MDS) (Stresemann and Lyko, 2008). Once incorporated into DNA, these nucleoside analogues, act as suicide inhibitors. Normally, DNMTs catalyze methylation by forming a covalent bond between a catalytic cysteine residue and the C6 position of cytosine, enabling the transfer of a methyl group from S-adenosyl-L-methionine (SAM) to C5 position of the base. The intermediate is resolved through β-elimination, a step in which the removal of a proton from the newly methylated C5 position initiates an electron cascade that breaks the covalent bond between the enzyme’s cysteine residue and the C6 position of cytosine, thereby releasing the active enzyme (Stresemann and Lyko, 2008). However, in DNA containing azacytosine, a nitrogen substitution at the C5 position blocks this critical release step. As a result, the DNMT becomes irreversibly locked onto the DNA (Stresemann and Lyko, 2008). Loss of active DNMTs prevents maintenance of methylation patterns during replication, causing passive, genome-wide demethylation and reactivation of silenced tumor suppressor genes. Thus, like topoisomerase poisons, azacytidine and decitabine exert their effects by generating covalent DPCs that are both directly cytotoxic and have profound downstream consequences for the epigenome (Ruggiano and Ramadan, 2021).

Unlike the “suicide inhibitors” described above, platinum-based drugs (such as cisplatin, carboplatin, and oxaliplatin) induce DPCs indirectly. Rather than targeting an enzyme, these drugs act as direct DNA-damaging agents. They form covalent “adducts,” primarily linking adjacent guanine bases. These platinum-DNA adducts twist the DNA helix into a distorted structure. This distortion acts as a magnet for various cellular proteins, such as High-Mobility Group (HMG) box proteins and transcription factors. Once these proteins bind to the damaged DNA, they become trapped, forming bulky DPCs that physically block DNA replication and transcription (Jamieson and Lippard, 1999; Kartalou and Essigmann, 2001).

**FIGURE 2:**
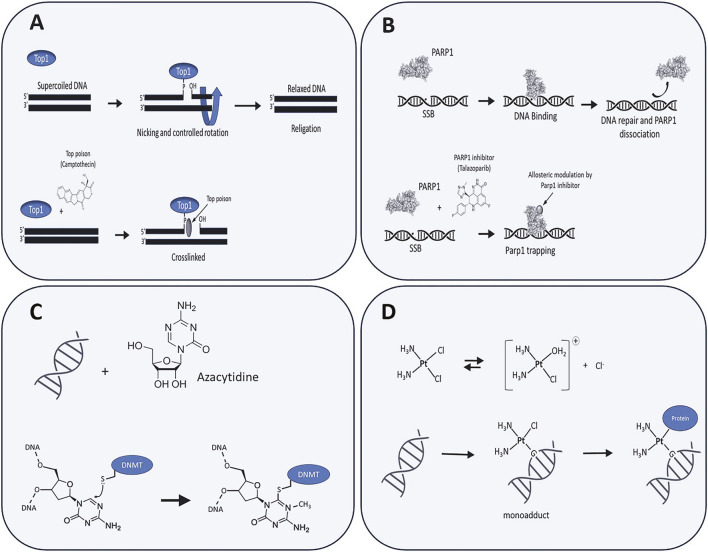
Mechanisms of Chemotherapy-Induced DNA-Protein Crosslinks (DPCs). Schematic illustration of the distinct molecular mechanisms by which four major classes of anticancer agents generate DPCs. **(A)** Topoisomerase Poisons. These agents interfere with the catalytic cycle of topoisomerases. For example, Topoisomerase I (TOP1) poisons like camptothecin stabilize the transient covalent complex formed between the enzyme and DNA during the relaxation of supercoiling. By inhibiting the re-ligation of the single-strand break, they trap TOP1 on the DNA backbone, as depicted. Similarly, Topoisomerase II (TOP2) poisons, such as etoposide, function by trapping TOP2 on DNA at double-strand breaks. **(B)** PARP1 Inhibitors. Poly (ADP-ribose) polymerase 1 (PARP1) inhibitors, such as talazoparib, bind to PARP1 at sites of single-strand breaks (SSBs). This binding allosterically modulates the enzyme, trapping it on the DNA and creating a stable DPC-like obstruction that blocks DNA repair and replication. **(C)** Azacytidine. This hypomethylating agent, a nucleoside analog, is incorporated into DNA during replication. It acts as a pseudosubstrate for DNA methyltransferase (DNMT), which becomes covalently and irreversibly entrapped upon attempting the methylation reaction. **(D)** Cisplatin. Platinum-based agents first form a reactive monoadduct with DNA. This adduct can subsequently crosslink with an adjacent protein in a non-enzymatic and non-specific manner.

A third class of therapeutically relevant DPCs involves stalled RNA polymerases (RNAPs). Unlike topoisomerases or DNMTs, RNAPs interact with DNA non-covalently. However, they can become stably stalled at sites of DNA damage, topological stress, or transcription-replication conflicts, where they act as large, obstructive complexes that disrupt transcription and activates DNA damage signaling (Damsma et al., 2007; Wang et al., 2010). This demonstrates that therapeutic protein trapping is a broad pharmacological principle, where agents ranging from hypomethylating drugs to platinum compounds generate physical obstructions-whether through irreversible enzyme suicide inhibition, adduct-mediated trapping, or steric stalling-to disrupt genomic processes. This pharmacological diversity is visually summarized in Figure 2, which contrasts the distinct trapping mechanisms of topoisomerase poisons, PARP inhibitors, azacytidine, and platinum agents. By mapping these drug-specific interactions, the figure underscores how diverse classes of anticancer therapies converge on the common strategy of creating toxic, chromatin-bound protein obstacles.

### 1.4 Consequences at the replication fork and toxicity

The replication fork is where the damaging consequences of trapping are most evident. Stable protein-DNA adducts act as severe physical impediments to the replication machinery, causing fork stalling, collapse, or reversal into fragile “chicken foot” structures that are prone to nuclease degradation (Westhorpe et al., 2024; Van Ravenstein et al., 2022). Collapsed forks frequently generate DNA double-strand breaks (DSBs), while prolonged stalling can activate dormant origins, both of which amplify replication stress and activate the ATR-mediated DNA damage response. If unresolved, this cascade of events leads to chromosomal rearrangements and cell death, which is the key mechanism underlying the therapeutic efficacy of DPC-inducing agents (Saxena and Zou, 2022).

While this replication-dependent toxicity is essential for killing tumors, it simultaneously damages healthy, rapidly proliferating tissues, particularly hematopoietic (blood-forming) progenitors. This leads to “dose-limiting” toxicities-primarily myelosuppression-which creates a very narrow window for safe dosing. This principle is clearly illustrated by PARP inhibitors: potent trappers like talazoparib are far more effective than weaker trappers like veliparib, but this increased potency comes with more severe side effects, such as neutropenia, thrombocytopenia, and anemia (Murai et al., 2012; Muvarak et al., 2016). Such toxicities occur because rapidly dividing hematopoietic stem and progenitor cells are especially vulnerable to replication stress from DPCs.

This clinical challenge has driven the search for next-generation drugs designed to uncouple therapeutic efficacy from off-target toxicity. One significant advance is the development of selective PARP1 inhibitors, such as saruparib (AZD5305). By maintaining potent PARP1 trapping while avoiding the inhibition of PARP2, this agent mitigates the severe hematological side effects associated with earlier dual inhibitors (Illuzzi et al., 2022).

A parallel strategy involves Antibody-Drug Conjugates (ADCs), such as trastuzumab deruxtecan. These agents use a monoclonal antibody to deliver a potent topoisomerase poison directly to tumor cells expressing a specific surface antigen (e.g., HER2). This maximizes the drug concentration at the tumor site while minimizing systemic exposure (Linehan et al., 2021). Ultimately, while the replication stress driven by DPC-induced fork collapse is central to tumor cell death, it simultaneously imposes significant off-target toxicity on healthy proliferating tissues, necessitating the development of more selective targeting strategies.

### 1.5 The influence of DNA context on protein trapping

The formation and stability of trapped complexes are not uniform across the genome; they are heavily influenced by the local DNA sequence and structure. Key determinants include primary sequence features, the transcriptional status of the gene, the epigenetic landscape, and the higher-order chromatin architecture. Specific DNA sequences and secondary structures, like G-quadruplexes or hairpins, can hinder DNA replication or transcription, increasing the likelihood of protein stalling and trapping at these sites (Schiavone et al., 2014). Topoisomerase activity is often enriched in GC-rich regions and at non-B DNA structures like G-quadruplexes (G4), which can form in the promoters of many oncogenes. These structures can be difficult to replicate and are prone to forming stable trapped complexes. Trapping is also particularly toxic at sites of active transcription and at replication forks, as the collision of polymerases with a trapped complex is the primary mechanism for converting the lesion into a DSB. Transcriptionally active regions are particularly susceptible, as collisions between the transcription machinery and DNA damage can lead to RNAPII stalling, especially when lesions occur on the template strand. Notably, stalling of RNA polymerase II (RNAPII) arrest is more severe when DNA damage is positioned on the nucleosome-facing side of DNA, indicating that chromatin structure can mask DNA lesions from repair detection (Pestov et al., 2015).

The local chromatin state is a critical determinant of DPC dynamics. Tightly packed heterochromatin acts as a barrier, restricting the access of repair machinery; this can stabilize trapped proteins and delay their removal. In contrast, euchromatin-marked by histone acetylation and an open structure-allows for easier access, facilitating the faster recognition and resolution of trapped complexes (Hauer and Gasser, 2017). Moreover, the precise location of a DNA lesion relative to a nucleosome also plays a key role. Damage occurring within the nucleosome core is less accessible to repair factors than damage in the more exposed linker DNA.

Finally, DNA methylation plays a direct role in protein binding and trapping. For instance, DNMT1 exhibits a strong affinity for hemimethylated CpG sites during replication. This trapping is further enhanced when DNA is modified with cytosine analogs like 5-aza-dC, which covalently lock DNMTs to the DNA and prevent their release (Patel et al., 2010; Frauer and Leonhardt, 2009). Collectively, these findings demonstrate that the local DNA environment-both at the level of primary sequence and higher-order chromatin organization-plays a central role in determining the extent and consequences of protein trapping on DNA. Therefore, the formation, stability, and repair accessibility of trapped protein complexes are not uniform but are dictated by the local DNA sequence, transcriptional activity, and the compaction state of the surrounding chromatin.

### 1.6 Proteolytic debulking and extraction as the first steps of DPC repair

Cells have evolved a sophisticated and multi-layered defense network to resolve DPCs, initiating repair through a logical hierarchy that first addresses the physical obstruction posed by the protein adduct. This process begins with debulking pathways designed to reduce the steric hindrance of the DPC, followed by precise enzymatic steps to restore the integrity of the DNA backbone. Cells frequently initiate DPC repair by breaking down the protein component into short peptides. The 26S proteasome has a well-documented role in clearing trapped topoisomerase II-DNA cleavage complexes (TOP2ccs) (Yan et al., 2016). During DNA replication, a more specialized pathway is activated involving the DNA-dependent metalloprotease SPRTN, which is recruited to stalled replication forks to selectively cleave the protein moiety of DPCs, including trapped PARP1 and TOP3A complexes, facilitating fork progression and maintaining genome stability (Saha et al., 2023a; Saha et al., 2021). Crucially, this proteolytic debulking does not fully restore the DNA template since it leaves a peptide remnant that continues to block high-fidelity replicative polymerases. Consequently, the repair process is tightly coupled with DNA damage tolerance mechanisms, requiring a polymerase switch to Translesion Synthesis (TLS) polymerases to bypass the peptide remnant and allow replication to proceed prior to final excision (Stingele et al., 2017).

For non-covalently but tightly bound protein complexes, such as those involving PARP1, a signal-driven extraction mechanism is employed. This process is initiated by SUMOylation of the trapped protein by the E3 ligase PIAS4, which is then recognized by SUMO-targeted ubiquitin ligases like RNF4. These ubiquitin chains serve as a recruitment signal for the AAA + ATPase segregase p97/VCP, which, in conjunction with its cofactors UFD1-NPL4, mechanically unfolds and extracts the ubiquitylated protein from chromatin (Mirsanaye et al., 2024). Accumulating evidence indicates that SUMOylation plays a multifaceted role in DNA-protein crosslink (DPC) repair that extends well beyond its canonical function as a simple signal for ubiquitin-mediated proteasomal degradation. This post-translational modification acts as a multifunctional regulatory hub, governing the spatiotemporal dynamics of topoisomerases under both physiological and genotoxic conditions (Pommier et al., 2022; Flotho and Melchior, 2013; Sun et al., 2022).

In unperturbed cells, SUMOylation is critical for regulating topoisomerase localization and activity; for example, it directs Topoisomerase IIα (TOP2α) to centromeres to ensure faithful chromosome segregation during mitosis and modulates Topoisomerase I (TOP1) interactions with the transcriptional machinery to suppress harmful R-loop formation (Azuma et al., 2005; Yoshida et al., 2016; Li et al., 2015).

In the context of DPC repair, SUMOylation operates as a molecular scaffold that contributes to the early recognition, spatial organization, and processing of lesions. As comprehensively discussed by Sun et al., DPC induction rapidly triggers SUMO-2 and SUMO-3 modification, creating a SUMO-rich environment essential for downstream repair (Borgermann et al., 2019). Critically, beyond recruitment, SUMOylation functions as a key regulator of DPC relocalization within the nucleus. Since the nucleolus lacks the necessary proteasomal machinery for efficient repair, SUMOylation promotes the essential export of trapped TOP1-DPCs from the nucleolus to the nucleoplasm (Rallabhandi et al., 2002). This relocalization exposes the lesion to nucleoplasmic repair complexes, allowing these SUMO chains to recruit RNF4, which ubiquitinates topoisomerase-DPCs in a PIAS4-dependent manner and promotes their proteasomal degradation (Sun et al., 2020a). Together, these findings support an expanded model in which SUMOylation stabilize protein–protein interactions at sites of damage, coordinating the recruitment of DPC repair factors such as the segregase p97/VCP and the metalloprotease SPRTN, restricting homologous recombination and thereby preventing deleterious double-strand breaks (Sun et al., 2022; Ruggiano et al., 2021). Notably, experimental studies have also demonstrated that SUMO modification of topoisomerase–DNA adducts facilitates downstream repair events independently of, or in parallel with, ubiquitin signaling pathways (Borgermann et al., 2019; Sun et al., 2020a). Additionally, the SUMO E3 ligase ZATT (ZNF451) functions in a non-proteolytic “remodeling” pathway. ZATT binds to and SUMOylates trapped TOP2, inducing a conformational change that exposes the TOP2-DNA junction, priming the DPC for direct hydrolysis by TDP2 (Sun et al., 2020b).

Following the removal of the bulk protein component, a peptide remnant often remains covalently linked to the DNA via a phosphotyrosyl bond. Two specialized enzymes resolve these terminal adducts: Tyrosyl-DNA Phosphodiesterase 1 (TDP1) hydrolyzes 3′-phosphotyrosyl linkages from trapped Topoisomerase I complexes, and Tyrosyl-DNA Phosphodiesterase 2 (TDP2) cleaves 5′-phosphotyrosyl bonds from trapped Topoisomerase II or TOP3A complexes (Saha et al., 2023a). Emerging evidence indicates that SPRTN and p97 function within an adaptor-mediated repair module rather than independently; the adaptor TEX264 binds TOP1 cleavage complexes and recruits both p97 and SPRTN to the trapped adduct to coordinate unfolding and proteolysis (Fielden et al., 2020). If these pathways fail, nucleolytic excision pathways provide a rescue. The MRE11 nuclease can excise the DNA segment containing the adduct, while the Nucleotide Excision Repair (NER) machinery (XPG, XPF-ERCC1) can perform dual incisions flanking the DPC (Saha et al., 2023a; Zhang et al., 2020; Sun et al., 2020c).

**FIGURE 3:**
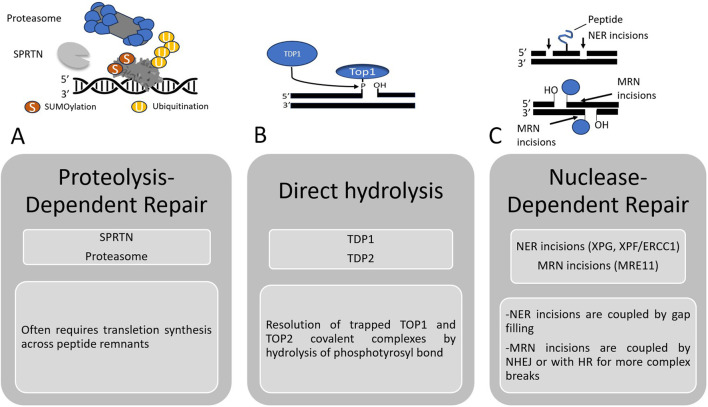
Overview of the Major DNA-Protein Crosslink (DPC) Repair Pathways. Illustration of the three principal mechanisms for the cellular repair of DNA-protein crosslinks (DPCs). **(A)** Proteolysis-Dependent Repair. This pathway removes the bulky protein component of the DPC through degradation. The covalently bound protein is often marked for proteolysis by post-translational modifications such as SUMOylation (S) and ubiquitination (U). This targets the protein for degradation by the proteasome or the specialized metalloprotease SPRTN. This process resolves the steric hindrance by reducing the DPC to a small peptide remnant still attached to the DNA, which is subsequently bypassed or removed by downstream processes, often involving translesion DNA synthesis. **(B)** Direct Hydrolysis. This specialized pathway acts on DPCs formed by Topoisomerase (TOP) enzymes, specifically TOP1 and TOP2. The enzymes Tyrosyl-DNA Phosphodiesterase 1 (TDP1) and Tyrosyl-DNA Phosphodiesterase 2 (TDP2) directly hydrolyze the phosphotyrosyl bond that links the TOP enzyme to the 3′and 5′DNA ends, respectively. This releases the protein and restores the integrity of the DNA backbone in a direct, one-step reaction. **(C)** Nuclease-Dependent Repair. This pathway excises the DPC along with the surrounding DNA segment. Two main nuclease systems are involved. The NER machinery, including the endonucleases XPG and XPF-ERCC1, makes incisions on the same DNA strand upstream and downstream of the DPC. The resulting single-strand gap is then filled in by DNA synthesis. The MRE11 nuclease, part of the MRE11-RAD50-NBS1 (MRN) complex, can make incisions that often lead to a DNA double-strand break (DSB) at the DPC site. These breaks are subsequently repaired by canonical DSB repair pathways such as Non-Homologous End Joining (NHEJ) or, for more complex breaks often encountered during replication, Homologous Recombination (HR).

Figure 3 provides a comprehensive map of these repair hierarchies-proteolysis, hydrolysis, and nucleolytic excision-emphasizing the redundant salvage pathways that tumor cells may exploit to survive DPC-inducing therapies when primary repair mechanisms are overwhelmed.

Recent work has also uncovered specialized pathways tailored for distinct lesion contexts, particularly those interfering with transcription. A dedicated pathway ensures the removal of DPCs that block RNA Polymerase II (RNAPII) on the transcribed strand, engaging the Transcription-Coupled Repair (TCR) machinery. This repair mechanism is strongly dependent on two key factors: Cockayne Syndrome group B (CSB) and Cockayne Syndrome group A (CSA). Mechanistically, CSB functions as the initial sensor, promoting the recognition and stabilization of the stalled RNAPII complex at the lesion site. Following this recognition, CSB recruits CSA, which orchestrates the ubiquitin-dependent remodeling of the stalled complex; this remodeling is critical as it supports either the preservation or the degradation of the polymerase, depending on local chromatin and signaling cues (Carnie et al., 2024). Furthermore, Flap endonuclease 1 (FEN1) can cleave DNA flanking protein-DNA adducts when these lesions undergo PARP1-dependent ADP-ribosylation. PARylation at the DPC site generates a recruitment signal for FEN1, which incises the DNA adjacent to the cross-linked protein, providing an alternative route for resolving DPCs formed by formaldehyde or Topoisomerase II (Sun et al., 2025).

### 1.7 Neddylation and PARylation as modulators of proteasomal degradation

Recent studies highlight critical roles for post-translational modifications, particularly neddylation and PARylation, in regulating the repair of DNA-protein crosslinks (DPCs). The interplay between these modifications and ubiquitin-mediated degradation constitutes a critical regulatory axis that influences both the recognition and clearance of trapped complexes. The covalent trapping of TOP1 on DNA is initially sensed through PARP1-mediated signaling, which drives local poly (ADP-ribose) (PAR) synthesis. This PAR scaffold serves a dual function: it recruits downstream factors such as TDP1 to facilitate excision, while simultaneously prevent premature proteasomal degradation of TOP1-DPCs. This regulatory step suggests that PARG-mediated de-PARylation is required to enable efficient proteolytic processing (Sun et al., 2021; Fabian et al., 2024).

Parallel to this PAR-dependent regulation, the catalytic efficiency of the ubiquitin machinery is dynamically modulated by Neddylation. This regulation specifically governs the Cullin-RING E3 ligases (CRLs), a major superfamily of ubiquitin ligases characterized by a central “Cullin” scaffold protein that coordinates the enzymatic complex by structurally bridging the catalytic RING domain with a specific substrate receptor. The enzymatic activity of these CRLs is strictly dependent on the conjugation of the ubiquitin-like molecule NEDD8 to a specific lysine residue on the Cullin scaffold-a process known as neddylation. This regulatory principle is exemplified by the DCAF13-CRL4 complex, a specific CRL4 assembly utilizing Cullin-4. Neddylation of the Cullin-4 scaffold induces a conformational change that promotes the recruitment and ubiquitylation of TOP1 cleavage complexes (TOP1ccs) by DCAF13, thereby effectively coupling the ubiquitin-proteasome pathway to lesion clearance. Consequently, inhibition of neddylation reduces CRL activity, impairs TOP1 turnover, and sensitizes cells to TOP1 poisons (Sun et al., 2023; Meroni et al., 2022). This critical balance of ubiquitin dynamics extends beyond TOP1 to other topoisomerases such as TOP3B (Saha et al., 2020; Saha et al., 2023b). Thus, across different DPC substrates, the precise modulation of ubiquitin signaling-whether by PARylation checkpoints or deubiquitinase recruitment-determines whether a trapped protein is safely resolved or persists as a genotoxic lesion.

From a broader perspective, the cell appears to have evolved these two distinct modification systems to solve different repair challenges. While the SUMO-Ubiquitin axis functions as the primary signal marking the DPC for physical removal via the proteasome or p97, the Neddylation and PARylation pathways modulate the kinetics and specificity of this removal. Neddylation acts upstream, serving as an activation switch for the E3 ligases themselves, ensuring that powerful ubiquitin machinery is only active when necessary. Conversely, PARylation acts at the lesion site to coordinate timing, ensuring that degradation does not occur until downstream repair factors (like TDP1) are recruited. This strategy prevents errors that could destruct chromatin-associated proteins and ensures that proteolytic debulking is tightly coupled to the restoration of DNA integrity.

### 1.8 The diverse biochemical activities and structural features of the DPC repair machinery

The cellular machinery dedicated to resolving DNA-protein crosslinks (DPCs) comprises a sophisticated network of proteins with diverse biochemical activities and structural architectures that first recognize the lesion and subsequently recruit effector enzymes to remove it. The recruitment of effector enzymes to DPC sites is governed by a hierarchical signaling network where E3 ubiquitin ligases translate specific post-translational modifications into signals targeting the complex for degradation. Rather than serving as primary lesion sensors, these ligases exhibit strict specificity for modified DPCs.

**FIGURE 4:**
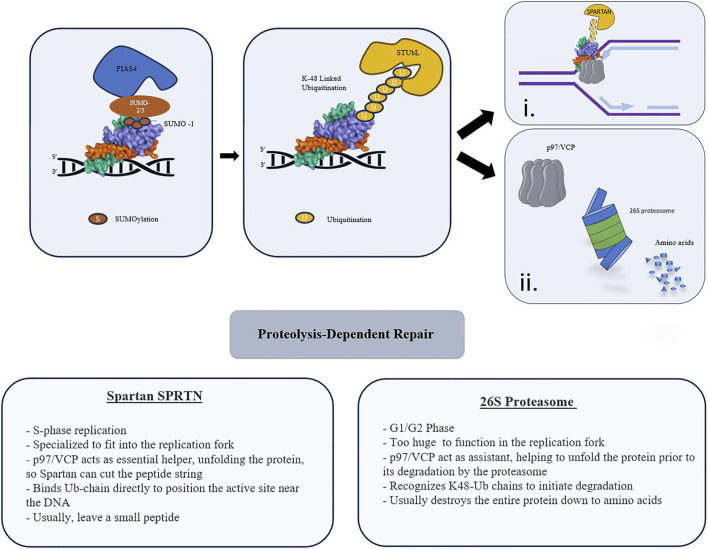
Proteolysis-Dependent Repair of DNA-Protein Crosslinks. Schematic representation of the SUMO-ubiquitin-dependent processing of DPCs mediated by p97 and SPRTN. The repair pathway initiates with the PIAS4-mediated conjugation of SUMO chains (SUMO-2/3 and SUMO-1) to the trapped protein. These modifications serve as a docking platform for SUMO-targeted ubiquitin ligases (STUbLs), which catalyze the formation of K-48 linked ubiquitin chains. The ATP-dependent segregase p97/VCP subsequently engages the ubiquitinated DPC to unfold or extract the crosslinked protein, facilitating downstream proteolysis via two distinct, cell-cycle-dependent pathways: (i) SPRTN-mediated proteolysis. During S-phase, the metalloprotease SPRTN is specialized to function within the restricted environment of the replication fork. It binds the ubiquitin chain and, with p97 assistance, cleaves the protein string, typically leaving a small peptide remnant. (ii) 26S Proteasome degradation. Primarily in G1/G2 phases, the 26S proteasome degrades the entire protein component into amino acids. This pathway relies on p97/VCP for substrate unfolding and extraction, as the proteasome complex is too large to access sterically hindered sites such as the replication fork.

A canonical mechanism involves the recognition of SUMOylated proteins by SUMO-Targeted Ubiquitin Ligases (STUbLs). For topoisomerase-derived DPCs, PIAS4 assembles SUMO-2/3 chains capped by SUMO-1. These polymers dock the STUbL RNF4, which catalyzes K48-linked ubiquitination to commit the adduct to proteasomal destruction. Figure 4 delineates the proteolysis-dependent repair module, explicitly highlighting the critical node of SUMO-ubiquitin signaling that coordinates p97 and SPRTN. This visualization underscores the therapeutic rationale for developing p97 and SPRTN inhibitors, as disrupting this specific extraction process forces cancer cells to accumulate lethal DPCs.

Distinct from this SUMO-dependent pathway, other ligases such as Cullin-RING Ligases (CRLs) and TRIP12 are recruited via alternative signaling contexts, frequently downstream of PARP1 activation, ensuring that a diverse array of substrates can be targeted for removal (Yang et al., 2021; Leng and Duxin, 2022).

Central to replication-coupled repair is the E3 ligase TRAIP, which binds the replisome and functions as a critical regulatory factor in the repair process (Wu et al., 2019). More specifically, by ubiquitylating the CMG helicase upon blockage, TRAIP dictates whether to recruit the p97 ATPase for extraction or the metalloprotease SPRTN for direct degradation (Deng et al., 2019). Crucially, while TRAIP-mediated polyubiquitylation is essential for p97 recruitment, SPRTN is distinctly activated by the physical extension of the DNA polymerase to the lesion and can degrade DPCs even in the absence of ubiquitylation, providing a rescue mechanism when signaling cascades are incomplete (Larsen et al., 2019).

Following this signaling cascade, effector proteins execute the physical removal of the adduct through proteolysis, mechanical extraction, or phosphodiester hydrolysis. Proteolysis-dependent repair focuses on reducing the bulk of the protein adduct. The most representative member of this class in humans is SPRTN, a specialized DNA-dependent metalloprotease containing an N-terminal SprT-like domain with a conserved HEXXH zinc-dependent catalytic motif and a Zinc-Binding Domain (ZBD). The primary role of the ZBD is to recognize and physically bind to DNA, specifically at or near the site of a DPC that has stalled a replication fork. The binding to DNA triggers a major conformational change in the protein that activates the separate SprT protease domain, thereby restricting proteolytic activity to sites of DNA damage (Perry and Ghosal, 2022; Li et al., 2019).

This replication-coupled proteolytic mechanism is evolutionarily conserved, originating from the yeast ortholog Wss1 (Weak suppressor of Smt3 protein 1), which functions as a first responder in proteolysis. Highlighting this functional homology, Stingele et al. (2014) identified that Wss1 possesses an analogous DNA-activated mechanism, where proteolytic activity is stimulated by DNA binding to cleave DPC components, including trapped Topoisomerase 1. However, while SPRTN regulation relies heavily on the DNA switch, Wss1 employs a “dual-switch” mechanism involving both ubiquitin and DNA binding to strictly confine activity to repair sites (Stingele et al., 2014). Cells lacking Wss1 accumulate DPCs and show marked chromosomal instability, underscoring that the reliance on a dedicated DNA-dependent metalloprotease is a fundamental, cross-species strategy for safeguarding genome integrity.

Expanding the proteolytic repertoire beyond metalloproteases, cells employ multiple DNA-dependent proteases to ensure robust DPC resolution. In yeast, the aspartic protease Ddi1 functions as a critical backup system. Serbyn et al. (2020) identified that Ddi1 is recruited in S-phase to persistent DPCs, such as stabilized Topoisomerase I cleavage complexes, particularly when the primary Wss1 and Tdp1 pathways are compromised. To this context, Ddi1 targets ubiquitylated DPCs and proteolytically removes the protein component, facilitating downstream repair independent of the 26S proteasome (Serbyn et al., 2020). In mammals, specialized non-proteasomal proteases like FAM111A preserve replication fork progression at protein obstacles. Distinct from SPRTN, the serine protease FAM111A employs a trypsin-like domain and requires a PCNA-interacting PIP-box and a specific protease site (S541) to remove TOP1-DPCs and non-covalent PARP1 complexes. Notably, its loss leads to TOP1cc accumulation and hypersensitivity to PARP inhibitors (Kojima et al., 2020). The evolution of this diversified proteolytic arsenal of metalloproteases, aspartic proteases, and serine proteases, likely reflects the biological necessity to tailor repair mechanisms to specific structural and temporal challenges. While the proteasome provides broad degradation capacity, specialized proteases like SPRTN and Wss1 offer the spatiotemporal precision required to resolve DPCs at the replication fork without to damage the surrounding chromatin, whereas factors like Ddi1 and FAM111A act as essential repair factors for distinct substrates or resistant lesions.

Complementing proteolysis is mechanical extraction, which utilizes ATP-dependent motors like the hexameric AAA + ATPase p97/VCP. The p97 complex features an N-terminal substrate recognition domain and two stacked ATPase domains (D1 and D2). Structural analysis reveals that the D1 domain is critical for hexamer stability and initial substrate engagement, while the D2 domain provides the primary ATP-hydrolyzing energy for protein unfolding; cycles of ATP hydrolysis in these domains drive major conformational changes that generate the mechanical force required to direct the substrate through the central pore for extraction. This process operates within a SUMO-ubiquitin-p97 axis, where SUMOylation flags chromatin-bound proteins for ubiquitination by STUbLs like RNF4, creating a handle for p97 recruitment (Sun et al., 2022; Krastev et al., 2022). This axis facilitates lesion clearance and modulates signaling, while p97 inhibition prolongs PARP1 trapping and sensitizes homologous-recombination-deficient tumor cells to PARP inhibitors. The p97 complex frequently operates with adaptor proteins such as TEX264, which recognizes TOP1 cleavage complexes and recruits both p97 and SPRTN, thereby linking ATP-driven extraction to proteolysis (Fielden et al., 2020).

Following debulking or extraction, the final step often involves direct hydrolysis of the covalent bond linking the protein remnant to the DNA backbone. Two representative members, TDP1 and TDP2, perform this function using distinct strategies. TDP1, a member of the phospholipase D superfamily, functions as a monomer to hydrolyze 3′-phosphotyrosyl bonds via a two-step mechanism involving a covalent enzyme-DNA intermediate. its active site contains two conserved HKD (histidine-lysine-aspartate) motifs, where a histidine residue acts as the nucleophile to attack the phosphotyrosyl bond (Kawale and Povirk, 2018; Dyrkheeva et al., 2020). In contrast, TDP2 belongs to the EEP superfamily and functions as a Mg^2+^-dependent dimer to hydrolyze 5′-phosphotyrosyl bonds in a single-step reaction, coordinating essential magnesium ions within its active site to activate a water molecule for the attack (Shimizu et al., 2023; Pommier et al., 2014). When direct hydrolysis is unavailable, cells resort to nucleolytic excision pathways. The MRE11-RAD50-NBS1 (MRN) complex can generate incisions at TOP2-DPCs, while the XPF-ERCC1 endonuclease provides a redundant pathway for excising TOP1-DPCs. This redundancy creates therapeutic opportunities, as inhibiting the TDP1/PARP axis induces synthetic lethality in tumors deficient in these backup nuclease pathways (Sun et al., 2020c).

In summary, the DPC repair machinery functions as a highly coordinated, multi-layered defense system. The modular architecture of E3 ligases ensures that diverse DPCs are precisely recognized and flagged for removal by structurally and mechanistically distinct effector enzymes. Table 1 categorizes these trapped complexes by their clinical relevance, linking specific chemotherapeutic agents (e.g., camptothecin, platinum drugs, PARP inhibitors) to the specific repair pathways that mediate drug resistance, thereby identifying potential targets for combination therapy.

**TABLE 1:** Categories of trapped protein–DNA complexes, their sources, and repair pathways.

Category	Representative proteins/Drugs	Nature of linkage	Endogenous/Exogenous source	Biological consequences	Major repair/Processing pathways
Covalent DNA–Protein crosslinks (DPCs)	Topoisomerases (Top1, Top2, Top3A); DNMTs (DNMT1, DNMT3A/B) with azacytidine/decitabine; PARP1 (with inhibitors), repair enzymes forming abortive intermediates (TDP1/2 substrates)	Covalent linkage between protein and DNA backbone (phosphotyrosyl, methylation-stalled complexes, azacytidine “suicide” adducts)	Endogenous: Enzymatic intermediates (Top1cc, Top2cc, DNMT methylation), aldehydes, formaldehyde, ROS. Exogenous: topoisomerase poisons (camptothecin, topotecan, irinotecan, etoposide), hypomethylating agents, platinum lesions, radiation.	Replication fork stalling, transcription blockage, replication-transcription conflicts, DSBs, chromatin distortion, genome instability	SPRTN/Wss1, p97/VCP (UFD1-NPL4), SUMO-RNF4, NER (XPC-XPA-XPG/XPF-ERCC1), MRN/MRE11, TDP1/2, ZATT–TDP2, CRL4-DCAF13, TEX264 recruitment, HR
Non-covalently trapped complexes	PARP1 (PARPi: Olaparib, talazoparib), RNA polymerase II (stalled at DNA lesions)	High-affinity but non-covalent DNA binding, stabilized by drugs or DNA damage	Endogenous: Transcription-replication conflicts, DNA lesions, exogenous: PARP inhibitors	Block of replication and transcription, accumulation of DNA breaks	TCR (for RNAPII); PARP1 removal via ubiquitin-proteasome system, replication-coupled clearance
Secondary/Indirect trapping	Histones; transcription factors; chromatin remodelers; HMG proteins (platinum-DNA binding), RNAPII stalling; PARP1 trapped by PARPi (talazoparib, olaparib, niraparib, AZD5305), aldehyde-crosslinked proteins	Non-covalent but stable trapping on damaged DNA or chromatin (drug-stabilized, lesion-induced, PARPi-induced, aldehyde-induced)	Endogenous: metabolic aldehydes, ROS, abasic sites, transcription stress. Exogenous: platinum drugs, PARP inhibitors, environmental aldehydes, UV distortions.	Chromatin distortion, transcriptional arrest, replication-transcription collisions, replication stress, impaired gene expression, DSB formation, fork collapse	Proteolysis-coupled repair (SPRTN, p97); transcription-coupled repair (CSA/CSB); RNAPII ubiquitin-proteasome degradation; PARP1 degradation via p97-SPRTN; NER; FA pathway, chromatin remodeling

Given the structural heterogeneity of these trapped complexes-ranging from covalent phosphotyrosyl bonds to non-covalent occlusions-effective resolution requires a highly specialized enzymatic arsenal. Table 2 summarizes the key repair enzymes and their regulation by post-translational modifications, while explicitly highlighting the therapeutic implications of each pathway. By linking molecular function directly to clinical vulnerabilities, this summary offers a framework for understanding how specific repair defects can be exploited for targeted cancer therapy.

**TABLE 2:** The biochemical functions of key repair factors.

Repair component/Enzyme	Primary function and mechanism	Regulation and signalling (PTMs)	Clinical relevance and therapeutic targeting
SPRTN (metalloprotease)	Proteolytic debulking: Cleaves the protein component of DPCs specifically during S-phase to prevent replication fork collapse.	Regulated by ubiquitination; activated by DNA binding (switch mechanism).	Mutated in Ruijs-aalfs syndrome (RJALS). Targetable by zinc-chelating inhibitors (e.g., hydroxamate scaffolds).
p97/VCP (AAA + ATPase)	Mechanical extraction: Unfolds and extracts ubiquitylated proteins from chromatin to facilitate proteasomal degradation.	Recruited via SUMO-Ubiquitin axis; relies on cofactors like UFD1-NPL4.	Inhibitors (e.g., CB-5339) trap ubiquitylated DPCs, leading to lethal DSBs; synergistic with DPC-inducing drugs.
TDP1/TDP2 (Phosphodiesterase’s)	Direct hydrolysis: TDP1 cleaves 3′-phosphotyrosyl bonds (TOP1); TDP2 cleaves 5′-phosphotyrosyl bonds (TOP2).	PARylation recruits TDP1; phosphorylation (CDK1) regulates TDP1 in mitosis.	TDP1 mutations cause SCAN1 neurodegeneration. Inhibiting TDP1 sensitizes tumours to topoisomerase poisons.
RNF4/TRAIP (E3 ubiquitin ligases)	Signalling and recruitment: RNF4 targets SUMOylated DPCs; TRAIP determines repair pathway choice (p97 vs. SPRTN) at replisomes.	SUMOylation serves as the priming signal for RNF4 (STUbL activity).	Critical for the resolution of drug-induced DPCs; defects lead to hypersensitivity to DNA-damaging agents.
Cullin-RING ligases (CRLs)	Targeted ubiquitination: DCAF13-CRL4 targets TOP1ccs for degradation.	Activated by neddylation of the cullin scaffold.	NAE inhibitors (pevonedistat) block neddylation, preventing DPC degradation and enhancing cytotoxicity of chemo.
26S proteasome	Complete degradation: Degrades bulk DPCs in G1/G2 phases (non-replication dependent).	Dependent on ubiquitination (K48-linked chains).	Proteasome inhibitors (e.g., bortezomib) can increase the burden of trapped protein complexes.

### 1.9 Modulation of DPC repair by cell cycle phase and local chromatin environment

The efficiency and choice of the DNA-protein crosslink (DPC) repair pathway are not static but are dynamically modulated by a host of cellular factors, particularly the cell cycle and the local chromatin environment. DPC repair is tightly linked to the cell cycle and especially during the S phase. The expression and activity of key DPC repair factors are often restricted to specific cell cycle phases. For instance, SPRTN levels are low or undetectable in non-replicating (Go/G1) cells, but become strongly expressed during the S phase, exactly when the replication is actively occurring. Moreover, SPRTN depletion leads to increased levels of DPCs specifically in S phase, indicating that its activity in replication -coupled repair is S phase dependent (Vaz et al., 2016). On the other hand, some repair activities are suppressed during mitosis to protect the integrity of condensed chromosomes. To this context, TDP1 is phosphorylated by CDK1 at serine 61 during mitosis with phosphorylation levels peaking in early mitosis and declining as cells transition into G1 phase. This modification promotes its dissociation from chromatin, ensuring that any persistent Top1ccs are instead handled by the specialized mitotic DNA synthesis (MiDAS) pathway, thereby preventing inappropriate repair attempts on highly condensed chromatin. A phosphodefective mutant of TDP1 (TDP1-S61A) fails to dissociate and remains persistently associated with mitotic chromosomes (Paul and Das, 2024).

The local chromatin environment profoundly influences DPC repair dynamics by controlling the accessibility of both the DNA lesion and the repair machinery. Histone post-translational modifications, such as ADP-ribosylation, acetylation, and methylation, play critical roles in modulating chromatin structure and directly impact the behavior of DNA-bound proteins.

For instance, histone ADP-ribosylation catalyzed by PARP1, has been shown not only to automodify PARP1, but also to modify nearby histones. This histone ADP-ribosylation is crucial for the timely release of PARP1 from DNA break sites, as it reduces the affinity of chromatin for PARP1 via electrostatic repulsion and loosens chromatin structure, allowing repair factors greater access. Consequently, the loss of histone ADP-ribosylation increases PARP1 trapping and sensitizes cells to PARP inhibitors (Zentout et al., 2024; Smith et al., 2023; Ozdemir et al., 2024).

Histone modifications such as acetylation and methylation play critical roles in modulating chromatin accessibility, thereby indirectly influencing the dynamics and trapping of DNA-associated proteins like PARP1, DNA methyltransferases (DNMTs), and RNA polymerases (RNAPs). Histone acetylation generally promotes a more open chromatin structure, facilitating access of DNA repair machinery and reducing the duration of protein trapping. For instance, histone deacetylase (HDAC) inhibitors have been shown to enhance PARP1 trapping at DNA double-strand breaks by increasing histone and PARP1 acetylation, which interferes with non-homologous end joining and sensitizes cells to PARP inhibitors (Robert et al., 2016). Similarly, the histone acetyltransferase KAT6A can form nuclear condensates that sequester PARP1, thereby reducing its trapping upon inhibitor treatment-an effect independent of its catalytic function (Zhan et al., 2024). Moreover, histone methylation also regulates PARP1 dynamics since PARP1 display preferential binding to chromatin marked by mono-methylated histones at active genes (Bamgbose et al., 2024).

Chromatin context also impacts the trapping and resolution of other nuclear proteins. The trapping of DNMTs using 5-aza-deoxycytidine is more persistent in compact chromatin (Patel et al., 2010). Similarly, RNAPII arrest at DNA lesions is stronger when damage occurs on nucleosome-facing surfaces, indicating that chromatin architecture can mask DNA damage from the repair machinery (Gerasimova et al., 2022). Open, euchromatic regions thus favor efficient recognition and resolution of protein-DNA complexes, while heterochromatic regions can delay repair by restricting access and stabilizing trapped proteins. Together, these findings underscore the importance of chromatin state-not only in determining DNA accessibility, but also in directly influencing the kinetic behavior and resolution of DNA-bound proteins during genomic stress.

From a clinical perspective, this dependence on chromatin context reveals a therapeutic opportunity to overcome drug resistance. Since open chromatin facilitates repair while compact heterochromatin stabilizes trapped complexes, epigenetic modulators such as HDAC inhibitors could be strategically deployed to alter the chromatin landscape. By increasing global acetylation, these agents may enhance the trapping efficiency of PARP inhibitors or topoisomerase poisons, effectively sensitizing tumors that rely on chromatin compaction to mask lesions from the replication machinery.

### 1.10 Genetic syndromes of defective DPC repair and their therapeutic implications

The critical role of DPC repair in maintaining genomic integrity is highlighted by human genetic disorders arising from germline mutations in key DPC repair factors, frequently mirrored by phenotypes observed in knockout mouse models.

These syndromes provide strong evidence that DPC repair is vital not only for eliminating drug-induced damage but also for eliminating the levels of endogenous DPCs generated by normal metabolic processes. The importance of DPC repair is particularly evident in long-lived, non-dividing cells like neurons, where the cumulative burden of unrepaired lesions can drive progressive neurodegeneration.

In humans, germline mutations in DPC repair genes are usually heterozygous resulting in partial loss of function and leading to specific disease phenotypes rather than embryonic lethality. Mutations in SPRTN lead to Ruijs-Aalfs Syndrome (RJALS) that is characterized by progeroid features, genomic instability, and a high incidence of early-onset hepatocellular carcinoma (Lessel et al., 2014; Ruijs et al., 2003). Similarly, TDP1 mutations cause Spinocerebellar Ataxia with Axonal Neuropathy (SCAN1), a progressive neurodegenerative disorder linked to defective resolution of topoisomerase I cleavage complexes (El-Khamisy et al., 2005).

Mouse models provide compelling complementary evidence for the indispensable nature of DPC repair. In contrast to the heterozygous mutations seen in human syndromes, complete knockout of essential genes such as Sprtn or Xrcc1 results in embryonic lethality, underscoring their non-redundant functions in protecting the genomes of rapidly proliferating cells (Demin et al., 2021).

The interplay between a patient’s genetic background and their response to chemotherapy has profound translational importance. Germline defects in DPC repair genes can render tumors hypersensitive to DPC-inducing agents, such as topoisomerase poisons and PARP inhibitors (PARPi). This heightened sensitivity, however, presents a significant clinical challenge, as it often extends to healthy tissues, leading to severe, dose-limiting toxicities that narrow the therapeutic window. Mechanistic insights from mouse studies further clarify these risks. For instance, the severe neurodegeneration observed in *Xrcc1*-deficient mice is largely rescued by the simultaneous deletion of *Parp1*. This finding demonstrates that the pathology is driven by the toxic trapping of PARP1, which cannot be properly resolved in the absence of XRCC1 trapping (Komulainen et al., 2021). This suggests that patients with XRCC1 deficiencies would not only be highly sensitive to PARPi but also exceptionally prone to severe neurological side effects (Demin et al., 2021).

Collectively, these findings from human genetics and animal models emphasize the necessity of considering a patient’s germline DPC repair status when designing treatment regimens with DPC-inducing agents. Such knowledge is crucial for predicting both therapeutic response and potential toxicities, paving the way for more personalized and safer cancer therapies. Translating this into practice requires integrating DPC-repair gene panels into routine pharmacogenomic screening. Detecting hypomorphic germline variants in genes like *SPRTN*, *TDP1*, or *XRCC1* could serve as a critical stratification tool-identifying not only “exceptional responders” who may benefit from lower therapeutic doses but also patients at high risk for severe, mechanism-based toxicities (such as neurodegeneration or myelosuppression) who require alternative regimens.

### 1.11 Alterations of the DPC repair machinery in cancer

Components of the DNA-protein crosslink (DPC) repair machinery are frequently altered in cancer through mutations, copy number variations (CNVs), or transcriptional silencing. These alterations create significant therapeutic vulnerabilities, offering opportunities to exploit the dependency of cancer cells on these pathways for survival.

**FIGURE 5:**
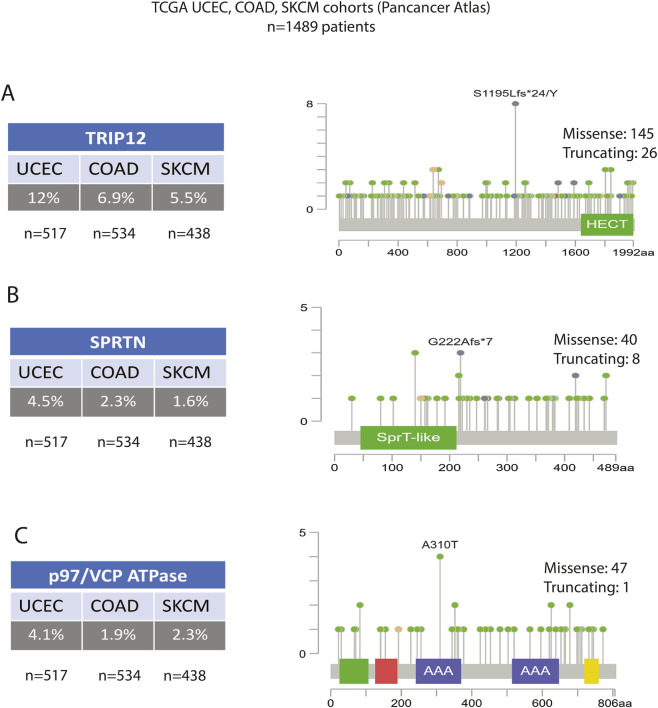
Genetic Alterations in Core DPC Repair Components in Human Cancers. Analysis of genetic alterations in key DNA-protein crosslink (DPC) repair genes across three cancer types with high mutational burdens: Uterine Corpus Endometrial Carcinoma (UCEC), Colon Adenocarcinoma (COAD), and Skin Cutaneous Melanoma (SKCM). Data is from The Cancer Genome Atlas (TCGA) Pancancer Atlas cohort (n = 1,489 patients). For each gene, the panel includes a table with the percentage of altered cases in each cancer type and a lollipop plot illustrating the distribution and type of mutations across the protein sequence. The vertical axis on each plot indicates the frequency of the mutation (number of patients). Green lollipops represent missense mutations, orange represents truncating mutations, and blue represents in-frame mutations. **(A)** TRIP12. The E3 ubiquitin ligase TRIP12 is the most frequently altered gene, with mutation rates of 12% in UCEC, 6.9% in COAD, and 5.5% in SKCM. The mutational spectrum includes a high proportion of truncating alterations, suggesting selection for loss-of-function. A recurrent frameshift mutation (S1195Lfs*24/Y) is noted within the catalytic HECT domain. **(B)** SPRTN. The DNA-dependent metalloprotease SPRTN is mutated in 4.5% of UCEC, 2.3% of COAD, and 1.6% of SKCM cases. Several inactivating mutations cluster within its SprT-like catalytic domain, including the recurrent G222Afs*7 frameshift mutation. **(C)** p97/VCP ATPase. The gene encoding the p97/VCP segregase is altered in 4.1% of UCEC, 1.9% of COAD, and 2.3% of SKCM cases. Mutations are distributed across the protein, including its two AAA + ATPase domains, with the A310T missense mutation being a notable recurrent alteration.

Our pan-cancer analysis of over 10,000 tumor samples from The Cancer Genome Atlas (TCGA) reveals that core components of the DNA-protein crosslink (DPC) repair machinery are genetically altered in approximately 5% of patients. This landscape is dominated by mutations in the E3 ubiquitin ligase TRIP12, which is altered in ∼3% of all cases. The mutational spectrum of TRIP12 includes a significant proportion of truncating and frameshift alterations distributed across the gene, suggesting a strong selection for loss-of-function. This is particularly relevant given that TRIP12 deficiency is linked to elevated PARP1 levels and may predict hypersensitivity to PARP inhibitors. In contrast, other key DPC repair factors are mutated at a much lower frequency. The tumor suppressor SPRTN is altered in just under 1% of cases, though these often include inactivating mutations within its catalytic domain. Other essential components, including TDP1, TDP2, VCP, and RNF4, each have alteration rates below 1%. Notably, these genetic alterations are not uniformly distributed and are significantly enriched in cancers with high mutational burdens, such as uterine corpus endometrial carcinoma (>12%), colorectal adenocarcinoma (∼7%), and cutaneous melanoma (∼5%), suggesting that DPC repair defects are a recurrent feature in genomically unstable tumors. As illustrated in Figure 5, the mutational landscape of DPC repair genes-particularly *TRIP12* and *SPRTN*-varies significantly across cancer types (UCEC, COAD, SKCM), providing a rationale for using these genetic signatures as predictive biomarkers for patient stratification in DPC-targeting therapies.

The work by Gatti et al. revealed that the expression level of the E3 ligase TRIP12 is a key determinant of PARP inhibitor (PARPi) sensitivity. In breast and ovarian cancer cohorts, low TRIP12 expression is negatively correlated with PARP1 protein levels, independent of BRCA status. Consequently, tumors with low TRIP12 tend to have higher PARP1 abundance, leading to enhanced PARP1 trapping upon PARPi treatment and increased cell death (Gatti et al., 2020). Conversely, in aggressive subtypes like triple-negative breast cancer (TNBC), high TRIP12 expression is often associated with lower PARP1 levels, which may confer resistance to PARPi and is linked to poorer survival (Krishnan et al., 2023). This suggests that TRIP12 expression could serve as a valuable predictive biomarker for stratifying patients for PARPi therapy. Beyond single-gene markers, these data support the development of a comprehensive ‘DPC Repair Signature’ for clinical stratification. As illustrated in Figure 5, the mutual exclusivity and tissue-specificity of mutations in *TRIP12*, *SPRTN*, and *p97* suggest that distinct tumor types rely on specific repair modules to survive therapy. Profiling these alterations could help oncologists predict which patients are most likely to benefit from synthetic lethal strategies, distinguishing those with innate hypersensitivity to PARP inhibitors from those harboring intact repair pathways that necessitate combination approaches.

### 1.12 Targeting DPC repair pathways for cancer therapy

A promising therapeutic paradigm in oncology is to exploit the reliance of cancer cells on their DNA-protein crosslink (DPC) repair machinery, either by directly inhibiting repair components or by combining DPC-inducing agents with other repair inhibitors to achieve synthetic lethality. This approach aims to overwhelm the cell’s repair capacity, leading to catastrophic genome instability and cell death.

Several combination strategies have shown significant potential. A prominent strategy involves pairing PARP inhibitors (PARPi) with topoisomerase I poisons like irinotecan; by suppressing the key single-strand break sensor PARP1, the repair of trapped Top1 cleavage complexes is prevented, leading to their collapse into lethal double-strand breaks during replication (Fabian et al., 2024; Znojek et al., 2014). Another approach combines PARPi with alkylating agents such as temozolomide or platinum-based compounds, where inhibiting PARP1 activity can hinder the overall repair of bulky adducts and enhance cytotoxicity (Gupta et al., 2014; Yusoh et al., 2025). A third key strategy pairs DPC-inducing agents with DNA Damage Response (DDR) inhibitors, particularly ATR kinase inhibitors, to create a powerful synthetic lethal effect by simultaneously increasing replication stress while dismantling the critical ATR-CHK1 checkpoint response (Zhang et al., 2023). These translational efforts are detailed in Table 3, which summarizes current clinical trials evaluating combinations of DPC-inducing agents with novel repair inhibitors (e.g., ATR, p97, and PARP inhibitors). The table highlights the progression from mechanistic biology to active clinical investigation, focusing on strategies designed to maximize synthetic lethality.

**TABLE 3:** Representative clinical trials of therapies targeting the DNA-protein crosslink response.

Trial (NCT/Name)	Phase	Cancer types enrolled	Drug/Regimen (mechanistic rationale)	Status/Early results (efficacy/Toxicity)
NCT04449562	Phase I	Advanced solid tumors and lymphomas	CB-5339 (oral p97/VCP inhibitor)	Early, dose-escalation safety study. Primary goal: safety/MTD; preliminary antitumor activity reported in early cohorts but no late-stage efficacy readout yet. Toxicity profile being defined.
NCT04644068/NCT06380751	Phase I/II	Advanced solid tumors; metastatic prostate; breast	AZD5305 (saruparib) - PARP1-selective inhibitor/trapper designed to preserve PARP1 trapping cytotoxicity but reduce PARP2-related hematologic toxicity. Studied as monotherapy and in combinations (hormone agents, chemo).	Ongoing. Designed to improve therapeutic window vs. older PARPi (reduced myelosuppression). Randomized phase II comparisons ongoing; early safety/pharmacology reported.
NCT02487095	Phase I/Ib	Advanced solid tumors; ovarian, small cell lung	Topotecan (TOP1 poison) + ATR inhibitor (VX-970/M6620/berzosertib)	Early phase: manageable safety at defined schedules; signals of activity in specific cohorts. These combos require careful dosing due to overlapping myelosuppression.
NCT03323034	Phase I	Pediatric/Adult solid tumors	Pevonedistat (NEDD8-activating enzyme inhibitor) + irinotecan (TOP1 poison) ± temozolomide	Phase I: safety/dose finding. Preclinical synergy reported; ongoing evaluation.
NCT01296763	Phase I/II	Colorectal, pancreatic, ewing sarcoma	Olaparib (PARPi) + irinotecan/topotecan/temozolomide	Several early trials completed: notable hematologic and GI toxicities when combined (dose-limiting); some durable responses in subsets but safety limits broad use; further optimization ongoing.
NCT02631733	Phase I	Advanced solid tumors	Veliparib + liposomal irinotecan combination -attempt to potentiate TOP1-cc cytotoxicity while modulating toxicity with liposomal formulation.	Phase I: dose-finding with manageable safety in early cohorts; combination development continues in selected indications.

Beyond these combinations, directly targeting the DPC removal machinery itself is an emerging approach. The DNA-dependent metalloprotease SPRTN is a key target. While SPRTN is a high-value therapeutic target, its inhibitors are currently in the preclinical stage of development. Lead compounds, such as Poloxin and other molecules based on hydroxamate scaffolds, function by targeting the catalytic core of the enzyme. SPRTN is a zinc-dependent metalloprotease that uses an essential zinc ion (Zn^2+^) within the conserved HEXXH motif of its SprT-like domain to cleave DPCs. These inhibitors act as zinc-chelating agents, binding directly to this ion within the active site (Li et al., 2019). This action physically obstructs and inactivates the enzyme, preventing the proteolytic removal of DPCs.

Another critical effector, the AAA + ATPase p97/VCP, represents a central hub for DPC resolution and is therefore an attractive therapeutic target. This complex acts as an ATP-dependent segregase, which mechanically unfolds and extracts ubiquitylated proteins from chromatin following their recognition by SUMO-targeted ubiquitin ligases like RNF4. The therapeutic strategy involves small-molecule inhibitors that target the enzyme’s ATPase domains (D1 and D2). By preventing ATP hydrolysis, these inhibitors lock p97/VCP in a static conformation, disabling the motor function required to generate mechanical force. This creates a critical bottleneck, causing ubiquitylated DPCs-which are already primed for removal-to accumulate on the DNA. The persistence of these bulky adducts leads to unresolved replication stress, fork collapse, and the formation of lethal double-strand breaks, thereby synergizing with DPC-inducing chemotherapies (Kilgas and Ramadan, 2023). While several p97/VCP inhibitors are in clinical development, their common analytical basis is the disruption of this essential protein extraction process.

Inhibitors of other DPC repair components, which, are also being explored to heighten DPC toxicity. For instance, inhibitors of DNA-PKcs, a critical enzyme in non-homologous end joining (NHEJ), such as peposertib (M3814) and nedisertib (M1774), block the repair of double-strand breaks arising from DPC-stalled forks, thereby increasing their cytotoxic burden (Zhang et al., 2023). Another strategy targets the NEDD8-activating enzyme (NAE) with inhibitors like pevonedistat (MLN4924). By blocking the neddylation pathway required for proteasome function, these drugs prevent the degradation of ubiquitylated DPCs, causing them to persist on the DNA (Ferris et al., 2020; Vanderdys et al., 2018).

Synthetic lethal strategies combining DPC-inducing drugs with ATR inhibitors, proteasome modulators, or SPRTN inhibitors are a promising avenue to further sensitize tumors with pre-existing DNA repair defects. Despite the clear mechanistic rationale, the clinical success of these combinatorial regimens is frequently hampered by overlapping toxicities. Since DPC-inducing agents target replication, they invariably affect rapidly dividing healthy tissues, particularly the hematopoietic system. Consequently, trials combining PARP inhibitors with chemotherapy or ATR inhibitors have often encountered severe dose-limiting myelosuppression, necessitating dose reductions that compromise antitumor efficacy. Furthermore, the emergence of acquired resistance-through mechanisms such as restoration of homologous recombination or drug efflux-remains a persistent barrier to durable responses. To decouple therapeutic efficacy from systemic toxicity, next-generation strategies are shifting toward precision targeting. First, the development of PARP1-selective inhibitors, such as saruparib (AZD5305), represents a major advance; by sparing PARP2, these agents maintain potent PARP1 trapping activity while significantly reducing the erythropoietic toxicity associated with first-generation dual inhibitors. Second, Antibody-Drug Conjugates (ADCs), like trastuzumab deruxtecan, exploit tumor-specific antigens to deliver high concentrations of topoisomerase poisons directly to the cancer cell. This “payload delivery” approach maximizes the formation of cytotoxic DPCs within the tumor while minimizing systemic exposure, thereby widening the therapeutic window.

A deeper mechanistic understanding of patient-specific repair capacities is essential for advancing these therapies. The use of functional biomarkers, such as the expression levels of TRIP12 or p97, could enable the rational selection of patients most likely to benefit from these regimens. Furthermore, high-resolution mapping techniques such as TOP1 CAD-seq, END-seq and SHAN-seq now allow for genome-wide analysis of DPC distribution and repair kinetics, offering a powerful platform to uncover locus-specific vulnerabilities (Kuzin et al., 2022; Tang et al., 2022; Morgan et al., 2024). Together, these insights will guide the design of new therapeutics that exploit the Achilles’ heel of cancer cells-their reliance on a fully functional DPC repair system to survive replication stress.

## 2 Conclusion

DNA-protein crosslinks (DPCs) represent a distinct class of DNA lesions that pose a profound threat to genomic integrity by physically obstructing fundamental processes such as DNA replication and transcription. To counteract this challenge, eukaryotic cells have evolved a sophisticated and highly coordinated defense network that integrates proteases, ATP-dependent segregates, nucleases, and phosphodiesterases into a flexible and partially redundant system. This network dynamically adapts to cell cycle stage, chromatin context, and upstream signaling cues to ensure timely lesion resolution. Dysregulation of DPC repair contributes to developmental disorders, neurodegeneration, and tumorigenesis, while simultaneously creating therapeutic vulnerabilities that can be exploited in cancer treatment.

The pharmacological stabilization of transient enzyme -DNA intermediates, particularly those involving topoisomerases and PARP, converts essential cellular factors into highly cytotoxic adducts and remains a cornerstone of modern cancer chemotherapy. Looking forward, a major research priority will be to refine this therapeutic paradigm to maximize clinical benefit. Achieving this goal will require strategies that expand the therapeutic window by enhancing tumor-selective cytotoxicity while limiting systemic toxicity, an effort that will be increasingly guided by biomarker-driven patient stratification. Predictive markers, such as TRIP12 expression, hold particular promise for identifying tumors with heightened dependence on DPC repair pathways and for informing rational treatment selection.

Future progress will also depend on a deeper mechanistic understanding of the regulatory complexity, spatiotemporal organization, and pathways choice that govern DPC repair in physiological and pathological contexts. Integrating these fundamental insights with emerging technologies for functional biomarker assessment and targeted drug delivery will be essential for translating DPC biology onto clinically actionable strategies. Collectively, these advances are expected to drive the development of more precise, personalized, and durable therapeutic interventions that exploit the intrinsic replication stress vulnerabilities of cancer cells.
